# BDNF haploinsufficiency induces behavioral endophenotypes of schizophrenia in male mice that are rescued by enriched environment

**DOI:** 10.1038/s41398-021-01365-z

**Published:** 2021-04-22

**Authors:** Mahmoud Harb, Justina Jagusch, Archana Durairaja, Thomas Endres, Volkmar Leßmann, Markus Fendt

**Affiliations:** 1 Institute for Pharmacology and Toxicology, Otto-von-Guericke University Magdeburg, Magdeburg, Germany; 2 Institute of Physiology, Otto-von-Guericke University Magdeburg, Magdeburg, Germany; 3 Center for Behavioral Brain Sciences, Otto-von-Guericke University Magdeburg, Magdeburg, Germany

**Keywords:** Learning and memory, Molecular neuroscience

## Abstract

Brain-derived neurotrophic factor (BDNF) is implicated in a number of processes that are crucial for healthy functioning of the brain. Schizophrenia is associated with low BDNF levels in the brain and blood, however, not much is known about BDNF’s role in the different symptoms of schizophrenia. Here, we used BDNF-haploinsufficient (BDNF^+/−^) mice to investigate the role of BDNF in different mouse behavioral endophenotypes of schizophrenia. Furthermore, we assessed if an enriched environment can prevent the observed changes. In this study, male mature adult wild-type and BDNF^+/−^ mice were tested in mouse paradigms for cognitive flexibility (attentional set shifting), sensorimotor gating (prepulse inhibition), and associative emotional learning (safety and fear conditioning). Before these tests, half of the mice had a 2-month exposure to an enriched environment, including running wheels. After the tests, BDNF brain levels were quantified. BDNF^+/−^ mice had general deficits in the attentional set-shifting task, increased startle magnitudes, and prepulse inhibition deficits. Contextual fear learning was not affected but safety learning was absent. Enriched environment housing completely prevented the observed behavioral deficits in BDNF^+/−^ mice. Notably, the behavioral performance of the mice was negatively correlated with BDNF protein levels. These novel findings strongly suggest that decreased BDNF levels are associated with several behavioral endophenotypes of schizophrenia. Furthermore, an enriched environment increases BDNF protein to wild-type levels and is thereby able to rescue these behavioral endophenotypes.

## Introduction

Schizophrenia is a neuropsychiatric disorder associated with life-long disabilities and a reduced life expectancy^1^. Typically, the symptoms appear in adolescence or early adulthood and include positive symptoms like hallucinations and delusions, negative symptoms like social withdrawal and lack of motivation, and cognitive symptoms like difficulties in memory, attention, and executive functions^1^. Schizophrenia is a neurodevelopmental disorder, caused by a combination of genetic susceptibility and environmental insults^2^. Furthermore, alterations in the inflammatory and immune systems are involved in the etiopathogenesis of schizophrenia^3,4^. All these factors induce or contribute to molecular, cellular, and structural changes in the brain, which eventually cause the symptoms of schizophrenia^5^.

Some neuropathological features of schizophrenia are correlated with reduced levels of brain-derived neurotrophic factor (BDNF). BDNF belongs to the protein family of neurotrophins and is secreted by neurons^6,7^. BDNF is involved in the survival, development, and differentiation of neurons and is crucial for synaptic plasticity^6,8–10^. A plethora of findings demonstrated a pivotal role of BDNF in learning and memory^11–15^. Studies using heterozygous BDNF-deficient mice and mice carrying the val66met BDNF polymorphism that reduces activity-dependent BDNF secretion^16^ show deficits in learning and memory and suggest that BDNF is involved in different neuropsychiatric disorders^17–21^. Several lines of evidence support an important role of BDNF in schizophrenia: first, schizophrenic patients show lower BDNF levels in the blood^22^ and cerebrospinal fluid^23^, as well as in different brain areas^24–27^. Second, BDNF blood levels of drug-naive first-episode patients are negatively correlated with positive symptoms^28,29^. Third, the single-nucleotide Val66Met polymorphism in the BDNF gene, which is considered to reduce activity-dependent BDNF secretion^16^, might be associated with increased susceptibility to schizophrenia, as well as with particular symptoms and the onset age of schizophrenia^30–35^ (but see ref. ^36^). Based on these and other findings, more research on how BDNF affects symptom presence and intensity and/or therapeutic responsiveness is demanded, with the final aim to also foster novel, BDNF-associated treatment approaches^30,37^.

A very useful animal model to investigate the role of BDNF in behavioral endophenotypes of schizophrenia is the BDNF-haploinsufficient (BDNF^+/−^) mouse strain that has an ~50% reduction in BDNF expression^38^. In mice, several behavioral endophenotypes of schizophrenia, such as sensorimotor gating deficits or impaired executive functions^39^, can be measured with experimental tests such as prepulse inhibition (PPI) of the startle response^40,41^ or the attentional set-shifting task (ASST), a measure for cognitive flexibility^42^. Of interest is also fear and safety learning^43,44^ since associative learning is impaired in schizophrenia patients^45,46^ and recent studies found deficient safety learning in these patients^47,48^. A previous study reported that PPI is not affected in young (13 weeks old) BDNF^+/−^ mice^49^. Regarding cognitive flexibility, reversal learning but not strategy shifting was impaired in young (8 weeks) BDNF^+/−^ mice^50^. However, several studies demonstrated that some behavioral deficits appear first in mature adult BDNF^+/−^ mice^51–54^. The latter is critical since mature adultness (3–6 months in mice, 20–30 years in humans^55,56^) is the usual age of the first admission for schizophrenia^28,29,57^. To the best of our knowledge, mature adult BDNF^+/−^ mice (≥5 months) were not tested for behavioral endophenotypes of schizophrenia so far.

Thus, the aim of this study was to investigate the role of BDNF in mouse behavioral endophenotypes of schizophrenia. Male mature adult (i.e., 5–6-months old) BDNF^+/−^ mice and their wild-type littermates (BDNF^+/+^ mice) were submitted to the following schizophrenia-relevant behavioral tests: (a) ASST as a measure for cognitive flexibility, (b) PPI of the acoustic startle response as a measure for sensorimotor gating, and (c) safety and contextual fear conditioning as measures of associative memory processes. Since behavioral deficits and BDNF brain levels in BDNF^+/−^ mice can be rescued by housing them in enriched environmental conditions^58,59^, the second set of mice was exposed to enriched environment (EE) for 2 months and subsequently submitted to the same behavioral tests. After completing all behavioral tests, BDNF levels of several brain areas were analyzed. Our hypothesis was that mature adult BDNF^+/−^ mice express behavioral endophenotypes of schizophrenia that can be prevented by EE exposure.

## Materials and methods

### Animals

In this study, we used male heterozygous BDNF knockout mice (BDNF^+/−^ mice, Bdnf^tm1Tbn^), which were generated by replacing a fragment of the BDNF protein-coding exon with a selection marker^38^. Constitutive BDNF knockout mice were independently generated in different laboratories with all strains showing an identical phenotype^38,60–62^ and are generally accepted mouse models to study the consequences of chronically reduced BDNF protein levels^51–53,63–69^. The BDNF^+/−^ mice from these independently generated mouse strains express ~50% of wild-type BDNF protein levels in all tested brain areas throughout their lives. Consequently, they show deficits in synaptic plasticity in the hippocampus, neocortex, and amygdala^38,65,70–74^, and age-dependent memory deficits, e.g., in hippocampus-dependent and amygdala-dependent learning tasks^51–53,63,67,75^.

The BDNF^+/−^ and BDNF^+/+^ mice used in the present study were offspring of BDNF^+/−^/BDNF^+/+^ breeding pairs from a line bred on a C57BL/6J background (>10 generations). The mice’s age at the beginning of the behavioral test was 5–6 months. The mice were kept in genotyped mixed groups of 2–6 animals per cage in a humidity- and temperature-controlled room (50–55%, 22 ± 2 °C) with a light cycle of 12 h on/off periods (lights on at 6:00 a.m.). Water and food were available ad libitum. All behavioral tests were carried out during the light phase (10:00 a.m.–4:00 p.m.). The experiments were performed in accordance with international ethical guidelines for the use of animals in experiments (2010/63/EU) and were approved by the local authorities (Landesverwaltungsamt Sachsen-Anhalt, Az.42505-2-1172 UniMD).

### Experimental procedure

**Fig. 1:**
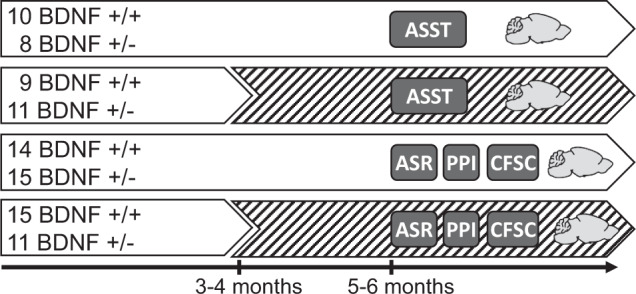
Experimental procedure. On the left, the group sizes and genotypes of the different batches of mice are given. White background indicates standard housing, hatched background enriched environment housing. On the bottom, the timelines of the experiment are shown. Experimental tests: ASST attentional set-shifting task, ASR acoustic startle response, PPI prepulse inhibition, CFSC contextual fear and safety conditioning. The brain symbol indicates the brain dissection after the end of the behavioral experiments.

The experimental procedure is outlined in Fig. 1. In total, 100 mice were used. Four different batches of mice were reared under standard housing conditions. At an age of 3–4 months, two of these batches were submitted to EE. Two months later, one batch of each housing condition was trained and tested in the ASST (standard housing: 10 BDNF^+/+^ mice, 8 BDNF^+/−^ mice; enriched environment: 9 BDNF^+/+^ mice, 11 BDNF^+/−^ mice). Since ASST was associated with food restriction, the body weight of these mice was controlled. The other batch of each housing condition was first tested for its startle reactivity and one day later for PPI (standard housing: 16 BDNF^+/+^ mice, 18 BDNF^+/−^ mice; enriched environment: 17 BDNF^+/+^ mice, 11 BDNF^+/−^ mice). Then, they were submitted to safety and contextual fear conditioning. Animal stress during the behavioral experiments was minimized by using minimally stressful stimuli, trained experimenters, and only short periods outside the home cage. After the behavioral experiments, mice were euthanized by decapitation under light isoflurane anesthesia and the brains were collected for the analysis of BDNF levels.

### Standard housing and enriched environment (EE)

Standard housing was performed in Makrolon Type III cages (38 × 22 × 15 cm) each equipped with one cardboard tunnel and paper tissue. EE housing took place in Makrolon Type IV cages (56 × 33 × 29 cm) each equipped with 2–3 cardboard tunnels, several paper tissues, wood gnawing blocks, and a mouse enclosure. For voluntary running, two running wheels (Fast-Trac, Plexx BV, Elst, The Netherlands) mounted on mouse igloos were placed in each cage. Every week, the cages were cleaned, the tissues renewed, and the enrichment objects newly arranged.

### Attentional set-shifting task (ASST)

Custom-built boxes (41 × 22 × 24 cm) consisting of a waiting area and—separated by transparent sliding doors—two further compartments (testing area) equipped with plastic bowls (5-cm diameter, 3-cm high) were used^76^. The bowls could be individually odorized and filled with different digging media. One particular odor or digging medium, respectively, was associated with a Choco Rice reward (ca. 20 mg, Nordgetreide GmbH & Co. K, Lübeck, Germany) underneath the digging medium. For the task, different exemplars of the stimulus dimensions “odor” and “digging medium” were used. The “odor” stimuli consisted of different odorants (e.g., citral, eucalyptol, s-(+)-carvone, R-( + )-carvone, valeric acid, and 2-phenylethanol, Sigma-Aldrich/Merck, Darmstadt, Germany) that were 1:20 dissolved in paraffin oil. In total, 30 µl of the solution were put on a small filter paper that was fixed on the bowls in the testing area. The “digging medium” stimuli consisted of wooden pearls of different sizes and colors (6- or 10-mm diameter, green, yellow, and brown; Aduis GmbH, Kiefersfelden, Germany). A further bowl with tap water was located in the waiting area.

Before the actual ASST, the mice were food-restricted (2 g food/day/animal) with the aim of reaching ca. 85% of the body weight with ad libitum feeding. Three days before the task, a habituation/prelearning period started. On the first day of this period, two bowls filled with bedding material and some reward were placed in the home cage of the mice. On the second day, all mice of a home cage were put into the experimental box equipped with the bowls (again filled with bedding material and some reward) and allowed to freely explore the box for 45 min. On the third day, the mice were individually put into the boxes. They had to collect rewards that were first put on the bedding material in the bowls but then gradually deeper in the bedding material with the aim that the mice learn to dig for the reward. The mice needed 30–60 min to learn the latter. On the next day, the last training stage occurred. Now, only one bowl was baited with reward (now always placed underneath the bedding material), and two different odorants were added to the two bowls. The mice now had to learn that one of two odorants predicted the reward (simple discrimination), however, the reward could be placed in both compartments (pseudorandomized from trial to trial). Each trial started with placing the mouse in the waiting area. Then, the sliding doors to the testing area were opened. The first two trials were free trials, i.e., the mouse had the possibility to dig in both bowls even if it dug in the unrewarded bowl first. From the third trial on, the sliding doors were closed as soon as the mouse started to dig in one bowl. Digging, retrieving, and eating the reward in the rewarded bowl was considered as a successful trial, digging in the unrewarded bowl as an unsuccessful trial. After each trial, the mouse was guided to the waiting area. Then, the two bowls were replaced by new bowls (reward location was randomized) and the next trial started. The simple discrimination phase was considered as passed if the criterion of six consecutive correct trials was made. Then, the ASST task immediately started. Two new odors were used and, importantly, a second stimulus dimension was now added: as digging material within the bowls, wooden pearls of different sizes and colors were used. In the subsequent phase, one of the two odors predicted the reward, while the digging material was irrelevant, i.e., not reward-associated (compound discrimination). Localization of the rewarded bowl in the two compartments was pseudorandomized. After reaching the criterion of six consecutive correct trials, the contingencies of the two odor cues were changed, i.e., the previously reward-associated odor was not reward-associated anymore, and vice versa (reversal 1). After reaching the criterion, mice were put back in the home cage. One day later, the next phase started (intradimensional shift). Two new odors and two new digging materials were used, however, still one of the odors was reward-associated while the digging materials were not relevant. After completion, contingencies were changed again (reversal 2). Then two new odors and two new digging materials were used but notably, now the digging material became relevant, i.e., reward-associated cue, whereas the odors became irrelevant (extradimensional shift). After completion, contingencies were changed a third time (reversal 3).

### Acoustic startle response and prepulse inhibition (PPI)

A startle system (SR-LAB, San Diego Instruments, San Diego, USA) with eight chambers (35 × 35 × 35 cm) was used for measuring the startle response and its inhibition by prepulses^77^. For the experiments, the animals were put inside transparent horizontal Plexiglas cylinders (4-cm diameter, 10-cm length) inside these chambers. Motion-sensitive transducers for detecting the startle response were mounted underneath the cylinders. The output signal of these transducers was digitized (sampling rate: 1 kHz) and stored on a computer. The mean transducer output 10–30 ms after startle stimulus onset was used as startle magnitude (arbitrary units). White background noise (55-dB SPL) was generated by high-frequency loudspeakers mounted in the center of the ceiling of the test chambers.

First, the mice were tested for their startle response to different startle stimulus intensities: after an acclimatization period of 5 min (only background noise), three blocks with eight trials each were presented. In each of the blocks, all eight possible stimulus intensities were presented in a pseudorandomized order (78-, 84-, 90-, 96-, 102-, 108-, 114-, and 120-dB SPL). The intertrial intervals were 30 s. One day later, PPI of the startle response was tested. After an acclimatization period of 5 min (only background noise), 12 startle stimuli were presented in order to habituate the animals. Afterward, six blocks with six trials each were presented. In each of the blocks, all six possible trial types were presented in a pseudorandomized order (startle stimulus without prepulse, startle stimulus with prepulses of 2, 4, 8, 12, or 16-dB SPL above background noise). All prepulses had a duration of 20 ms and preceded the startling stimulus by 100 ms (onset to onset). The intertrial intervals were 20 s. PPI for each prepulse intensity was calculated for each individual animal according to the following formula: PPI = (mean startle magnitude without prepulse – mean startle magnitude with prepulse)/(mean startle magnitude without prepulse/100).

### Safety and contextual fear conditioning

A computerized fear-conditioning system (TSE Systems, Bad Homburg, Germany) consisting of four identical transparent Perspex boxes (46 × 46 × 32 cm) was used. The boxes were surrounded by infrared animal detection sensor frames and located in a sound-attenuating chamber provided with loudspeakers for the acoustic stimuli (background noise of 55-dB SPL and the tone stimuli for safety conditioning), light sources (continuous illumination of ca. 10 lux), and a ventilation fan. The floor of the boxes consisted of removable stainless-steel grids (bars: 4-mm diameter, distance: 9 mm), which were connected to a shock unit and able to deliver foot shocks. Delivery of all stimuli was controlled by the TSE Fear Conditioning software. Movements of the animals were detected by the infrared sensors (distance: 14 mm). Freezing behavior was defined as no infrared beam crosses for more than 1 s. This automatic measurement of freezing in the TSE fear conditioning system was previously validated by demonstrating a high correlation with observer scoring of freezing^78,79^.

On the first day, mice were habituated to the conditioning boxes with a steel grid floor and the perspective safety CS (10-kHz tone, 85-dB SPL, 30 s). On the next two days, two safety conditioning sessions were performed^44^. The aversive unconditioned stimulus was a scrambled electric stimulus via the floor grid (0.4 mA, 2 s). In each of both conditioning sessions, the mice were exposed to five explicit unpairing of the tone stimulus (mean interval: 2 min, range: 1.5–2.5 min) and the unconditioned stimulus (Supplementary Fig. 1). Explicit unpairing means that the foot shocks were pseudorandomly presented between two tone presentations with a minimal time distance of 30 s to the previous and the next tone. With this protocol, the mice should learn that they can receive foot shocks in the conditioning context (i.e., contextual fear conditioning) but that the tone CS predicts a time when there will be no shock (i.e., cued safety conditioning). Thirty seconds after the last tone presentation, the mice were returned to their home cage. On the fourth day, a retention test of contextual fear and of conditioned safety was performed. The mice were placed into the conditioning boxes and after 30 s, 5 tone stimuli were presented at an interstimulus interval of 1 min.

### Brain dissections and BDNF measurements

Two weeks after the final behavioral experiment, the mice were anesthetized with isoflurane and euthanized by decapitation. The brains were removed and the prefrontal cortex, the dorsal hippocampus, the amygdala, and the nucleus accumbens were dissected, immediately deep-frozen, and stored at −80 °C until further analysis. For BDNF protein quantification, we used the Quantikine BDNF-ELISA kit (R&D Systems, Wiesbaden, Germany).

### Descriptive and analytical statistics

Group sizes were chosen based on power analyses using data from previous experiments or from literature. For statistics, GraphPad Prism 8.4 (San Diego, USA) was used. Normal distribution of the data was confirmed with D’Agostino & Pearson test and the equality of variances was checked with the Brown–Forsythe tests. Data are shown as box-and-whisker plots (box: median and quartiles, Tukey’s whisker). Statistical analysis was performed with multifactorial ANOVAs followed by Holm–Sidak’s multiple comparisons.

## Results

### Impaired performance of BDNF^+/−^ mice in the ASST is rescued by EE

**Fig. 2:**
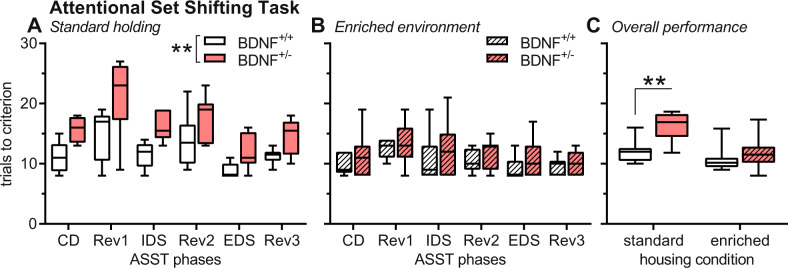
EE housing prevents the ASST performance deficits of BDNF^+/−^ mice. **A**–**C** Number of trials necessary to reach the criterion of six consecutive correct trials in the ASST, either depicted for the single phases of the task (**A**, **B**) or for the overall performance, i.e., the mean of all phases (**C**). (A) In all phases of the task, the number of trials to criterion was increased in BDNF^+/−^ mice after standard housing demonstrating an overall impairment. **B** After enriched environment, this difference between the genotype was not observed, indicating that the enriched environment rescues the performance deficits in BDNF^+/−^ mice (**C**). Group sizes: standard housing: 10 BDNF^+/+^ mice, 8 BDNF^+/−^ mice; enriched environment: 9 BDNF^+/+^ mice, 11 BDNF^+/−^ mice. **P < 0.01, main effects in ANOVA (**A**) or post hoc comparisons as indicated (**C**). CD compound discrimination, EDS intradimensional shift, IDS intradimensional shift, Rev reversal.

In Fig. 2, the mean number of trials to reach the criterion of six consecutive successful trials is depicted. After standard housing, BDNF^+/−^ mice needed more trials to complete the different ASST phases than BDNF^+/+^ mice (*F*_1,16_ = 20.23, *P* = 0.0004, Fig. 2A). There was also a significant effect of the ASST phases (*F*_5,80_ = 12.60, *P* < 0.0004) but no genotype × phase interaction (*F*_5,80_ = 0.59, *P* = 0.71). This indicates an overall, i.e., not phase-specific, deficit of standard-housed BDNF^+/−^ mice in the ASST.

After EE, the mice’s performance was not affected by the genotype (*F*_1,18_ = 1.08, *P* = 0.31, Fig. 2B). Again, the performance was different in the different ASST phases (*F*_5,90_ = 31.80, *P* = 0.0008) and there was no phase x genotype interaction (*F*_5,90_ = 1.62, *P* = 0.95). In addition, we analyzed the overall performance (Fig. 2C). This analysis revealed significant effects of genotype (*F*_1,34_ = 14.38, *P* = 0.0006) and housing condition (*F*_1,34_ = 16.68, *P* = 0.0003), as well as a genotype x housing condition interaction (*F*_1,34_ = 5.20, *P* = 0.03). Post hoc comparisons showed a significantly impaired overall performance in BDNF^+/−^ mice after standard housing (*t* = 4.18, *P* = 0.004) but not after EE housing (*t* = 1.10, *P* = 0.28). Of note, while overall ASST performance in BDNF^+/+^ mice was not affected by EE housing, performance in the reversal phases was improved by EE (*t* = 2.17, *P* = 0.045, see Supplementary Fig. 2).

These effects were fully supported by the analyses of the number of errors (see Supplementary Fig. 3). Taken together, ASST performance was impaired in BDNF^+/−^ mice, and this impairment was rescued by EE housing. Of note, neither the genotype nor enriched environment affected the body weight of the mice in this experiment (see Supplementary Fig. 4).

### Increased startle reactivity in BDNF^+/−^ mice is rescued by EE

**Fig. 3:**
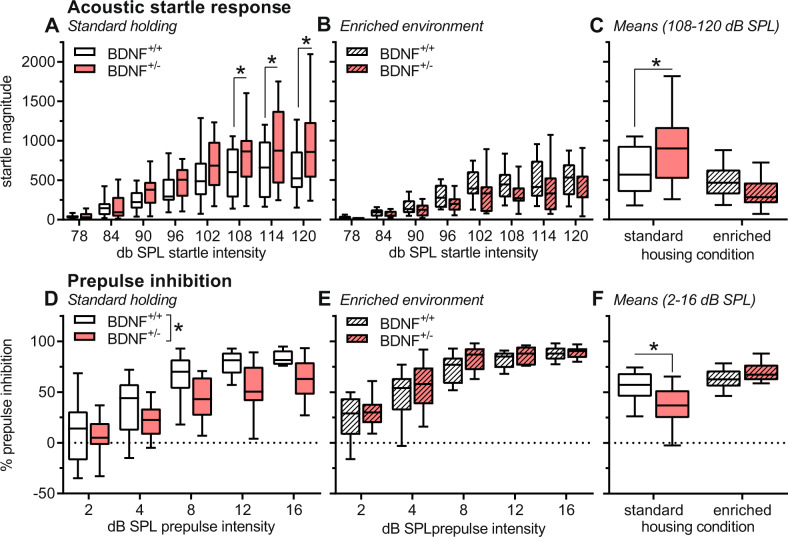
EE rescued increased startle reactivity and PPI deficits in BDNF^+/−^ mice. **A**, **B** Startle magnitudes using different startle stimulus intensities and (**C**) mean startle magnitudes (only startle stimuli with 108-, 114-, and 120-dB SPL). (A) Startle reactivity to high startle stimulus intensities was increased in BDNF^+/−^ mice after standard housing. **B** After enriched environment, startle magnitudes were not affected by genotype demonstrating (**C**) that enriched environment rescues the increased startle reactivity in BDNF^+/−^ mice. (D–E) Percent PPI (mean + SEM) using different prepulse intensities and (**F**) overall mean PPI. **D** PPI was reduced in BDNF^+/−^ mice after standard housing. **E** After enriched environment, PPI was not affected by genotype showing (**F**) that enriched environment rescued the PPI deficit in BDNF^+/−^ mice. Group sizes: standard housing: 14 BDNF^+/+^ mice, 18 BDNF^+/−^ mice; enriched environment: 14 BDNF^+/+^ mice, 11 BDNF^+/−^ mice. *P < 0.05, main effects in ANOVA (**D**) or post hoc comparisons as indicated (**A**, **C**, **F**).

We measured the startle magnitudes to acoustic stimuli with different intensities. Standard-housed BDNF^+/−^ mice had an exaggerated startle response at higher startle stimulus intensities compared to their BDNF^+/+^ littermates (Fig. 3A, interaction genotype x stimulus intensity: *F*_7,210_ = 2.14, *P* = 0.04). There was no main effect of genotype (*F*_1,30_ = 2.81, *P* = 0.10) but of stimulus intensity (*F*_7,210_ = 37.73, *P* < 0.0001). Post hoc comparisons showed higher startle magnitudes in BDNF^+/−^ mice at stimulus intensities of 108–120-dB SPL (*t*’s > 2.24, *P*’s < 0.03).

These differences were not observed if mice were EE-housed (Fig. 3B). Again, stimulus intensity had the main effect (*F*_7,175_ = 44.11, *P* < 0.0001) but there was neither a genotype effect (*F*_1,25_ = 2.45, *P* = 0.13) nor an interaction (*F*_7,175_ = 0.98, *P* = 0.45). This beneficial effect of EE was fully supported by an analysis including the data of both housing conditions (Fig. 3C). EE rescued the exaggerated startle response of BDNF^+/−^ mice observed in standard housing (interaction genotype x housing condition: *F*_1,55_ = 4.39, *P* = 0.04). In addition, EE decreased overall startle magnitudes, irrespective of genotype (*F*_1,55_ = 10.36, *P* = 0.002), whereas there was no main effect of the factor genotype (*F*_1,55_ = 1.02, *P* = 0.32).

### PPI deficits in BDNF^+/−^ mice are rescued by EE

In this test, the startle magnitudes to startle stimuli, preceded by prepulses with different intensities, were measured. With standard housing, PPI was impaired in BDNF^+/−^ mice (Fig. 3D). There were main effects of prepulse intensity (*F*_4,112_ = 83.75, *P* < 0.0001) and genotype (*F*_1,28_ = 6.04, *P* = 0.02) but no interaction between these two factors (*F*_4,112_ = 1.01, *P* = 0.40).

With EE, genotype did not affect PPI (*F*_1,23_ = 1.01, *P* = 0.32, Fig. 3E). Statistical analysis still showed an effect of prepulse intensity (*F*_4,92_ = 122.30, *P* < 0.0001), however, there was no interaction between these two factors (*F*_4,92_ = 0.50, *P* = 0.74). An additional analysis with data from both housing conditions (Fig. 3F) revealed that EE prevented the PPI deficit in BDNF^+/−^ mice observed after standard housing (interaction genotype x housing condition: *F*_1,51_ = 5.91, *P* = 0.02). EE generally increased PPI (*F*_1,51_ = 15.94, *P* = 0.002), whereas the genotype did not have main effects (*F*_1,51_ = 1.01, *P* = 0.30).

### Impaired safety learning in BDNF^+/−^ mice is rescued by EE

**Fig. 4:**
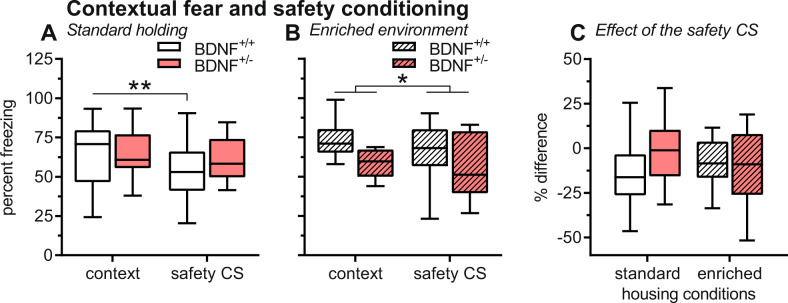
Conditioned contextual fear was not affected by genotype but its reduction by a safety CS was impaired in BDNF^+/−^ mice. This impairment was not observed after enriched environment. **A**, **B** Percent time spent freezing to the context and during presentations of the safety CS. **A** After standard housing, inhibition of freezing by the safety CS was observed in BDNF^+/+^ but not BDNF^+/−^ mice. **B** After enriched environment, both genotypes showed a reduction of freezing by the safety CS. **C** However, the analysis of the percent difference scores revealed neither an effect of genotype or housing condition nor an interaction. Group sizes: standard housing: 16 BDNF^+/+^ mice, 17 BDNF^+/−^ mice; enriched environment: 17 BDNF^+/+^ mice, 8 BDNF^+/−^ mice. **P < 0.01, *P < 0.05, main effects in ANOVA (**B**) or post hoc comparisons as indicated (**A**).

After safety conditioning, the mice were tested for their fear response to the conditioning boxes and their response to the safety CS. After standard housing, both genotypes expressed similar levels of freezing to the conditioning context (Fig. 4A, *F*_1,31_ = 0.59, *P* = 0.45) demonstrating successful contextual fear conditioning in both genotypes. During the safety CS, this freezing response was reduced in BDNF^+/+^ but not in the BDNF^+/−^ mice (interaction: *F*_1,31_ = 4.07, *P* = 0.05; safety CS: *F*_1,31_ = 11.74, *P* = 0.002) indicating impaired safety learning in BDNF^+/−^ mice. This was different after EE housing (Fig. 4B). There was a main effect of the safety CS (*F*_1,23_ = 6.76, *P* = 0.02) but neither genotype effects (*F*_1,23_ = 2.50, *P* = 0.13) nor an interaction between these factors (*F*_1,23_ = 0.10, *P* = 0.76). This suggests that EE rescued the impaired safety learning observed in BDNF^+/−^ mice after standard housing. For additional analysis, we calculated the percent freezing reduction by the safety CS (Fig. 4C): there were no effects of genotype and housing condition (F’s < 0.62, *P*’s > 0.61) and the interaction did not reach the level of significance (*F*_1,54_ = 2.49, *P* = 0.12).

### BDNF brain levels

Two weeks after the behavioral experiments, the mice were euthanized, the brains were dissected, and the BDNF levels in different brain areas were measured.

**Fig. 5:**
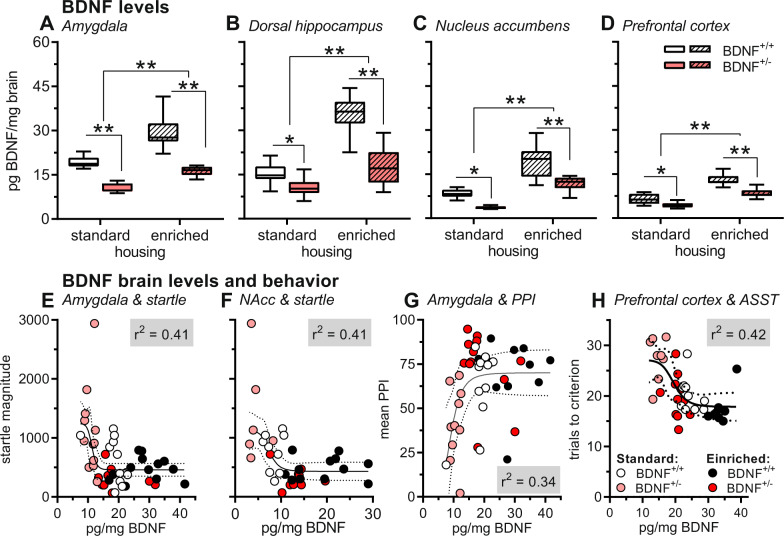
BDNF brain levels were generally decreased in BDNF^+/−^ mice, irrespective of housing condition. Exposure to enriched environment increased BDNF levels in both genotypes. Of note, BDNF^+/−^ mice previously housed in enriched environment had similar BDNF levels than standard-housed BDNF^+/+^ mice. This increase of BDNF brain levels in BDNF^+/−^ mice by enriched environment was associated with the rescue of behavioral deficits observed after standard housing. In wild-type mice, enriched environment increased BDNF brain levels but did not affect behavior. **A**, **D** Mean BDNF levels measured two weeks after the end of the behavioral experiments in the amygdala (**A**), dorsal hippocampus (**B**), nucleus accumbens (**C**), and prefrontal cortex (**D**). **P < 0.01, *P < 0.05, main effects in ANOVA or post hoc comparisons as indicated. **E**–**H** In some brain areas, individual BDNF brain levels were correlated with individual behavioral performance. We generally found that sigmoid regression curves best fitted the results. Depicted are the cases with *r*^2^-values >0.34. For example, higher BDNF levels in the amygdala and nucleus accumbens (NAcc) were associated with decreased startle magnitudes (**E**, **F**). In addition, PPI was increased with higher amygdaloid BDNF levels (**G**). In the ASST, the trials to criterion were reduced with higher BDNF levels in the prefrontal cortex (**H**). Group sizes (standard/BDNF^+/+^, standard/BDNF^+/−^, enriched/BDNF^+/+^, 8 enriched/BDNF^+/−^): amygdala: 13, 13, 18, 8; dorsal hippocampus: 13, 13, 17, 11; nucleus accumbens: 11, 6, 15, 11; frontal cortex: 10, 8, 8, 11.

In standard-housed BDNF^+/+^ mice, the total BDNF levels differed between regions with values of ca. 15–20 pg BDNF/mg brain tissue in the amygdala and the dorsal hippocampus and levels of ca. 5–10 pg BDNF/mg brain tissue in the nucleus accumbens and the prefrontal cortex (Fig. 5A–D). However, the effects of genotype and housing conditions were the same in all brain regions. BDNF levels were reduced in BDNF^+/−^ mice (*F*’s = 28.99, *P*’s < 0.0001) and EE housing increased BDNF levels (*F*’s = 63.25, *P*’s < 0.0001). For the amygdala and the dorsal hippocampus, the increase in BDNF levels after enriched environment was more pronounced in the wild-type mice (F’s > 8.17, *P*’s < 0.007), whereas no interaction between genotype and enriched environment effect was observed in the nucleus accumbens and the prefrontal cortex (*F*’s < 2.65, *P*’s > 0.11).

We analyzed whether individual BDNF levels in different brain areas were correlated with individual performance in the different tests. We generally found that sigmoid regression curves best fitted the results. This means that from a particular BDNF level on, no further behavioral changes were observed. In Fig. 5E–H, the brain areas with the best *r*^2^ values are depicted. These analyses showed that BDNF levels in the amygdala and nucleus accumbens were best correlated with the startle magnitude (*r*^2^ values > 0.41, Fig. 5E, F). In addition, the startle magnitudes were well correlated with BDNF levels in the prefrontal cortex (*r*^2^ = 0.38), while there was only a moderate correlation with BDNF levels in the dorsal hippocampus (*r*^2^ = 0.20, data not shown). For PPI, we found that BDNF levels in the amygdala correlated best with behavioral performance (*r*^2^ = 0.34, Fig. 5G), while BDNF levels in the prefrontal cortex and nucleus accumbens were only moderately correlated with PPI (*r*^2^ = 0.27 and 0.21, respectively, data not shown). There was no correlation between PPI and hippocampal BDNF levels (*r*^2^ = 0.05, data not shown). Furthermore, we observed only very poor correlations of BDNF levels with safety learning (amygdala: *r*^2^ = 0.03; nucleus accumbens: *r*^2^ = 0.13; dorsal hippocampus: *r*^2^ = 0.04; prefrontal cortex: *r*^2^ = 0.19, data not shown). Last, we found that BDNF levels in the prefrontal cortex were well correlated with overall performance in the ASST (*r*^2^ = 0.42, Fig. 5H).

## Discussion

Increasing evidence led to the hypothesis that the neurotrophin BDNF is involved in behavioral endophenotypes of schizophrenia^23–25,28–31,37^. The aim of the present study was to support and better understand this hypothesis utilizing BDNF^+/−^ mice. To this end, male mature adult BDNF^+/−^ mice were submitted to several behavioral tests relevant for schizophrenia to investigate whether genetically decreased BDNF levels lead to schizophrenia-typical behavioral impairments. After standard housing, we observed impaired cognitive performance in the ASST, exaggerated startle magnitudes, and PPI deficits, as well as impaired safety learning in BDNF^+/−^ mice. All these changes were rescued by EE housing of the mice. Analyses of the brain BDNF levels confirmed that BDNF^+/−^ mice have 30–60% less BDNF than their BDNF^+/+^ littermates. EE increased BDNF brain levels in both genotypes by 60–200%. This EE-induced recovery of BDNF levels in BDNF^+/−^ mice back to levels present in standard-housed BDNF^+/+^ animals suggests that the rescue of the behavioral impairments was based on the restoration of BDNF brain levels.

In contrast to many previous behavioral studies in BDNF^+/−^ mice, mature adults (5–6 months) instead of young adults' (2–3 months) BDNF^+/−^ mice were used here. This was based on previous data showing that particular behavioral impairments in BDNF^+/−^ mice are not present in young adults but in mature adult BDNF^+/−^ mice^51–53^. A second rationale for the use of this age was that the first admission for schizophrenia is usually during mature adultness^28,29,57^. At mature adultness and after standard housing, this study found several pronounced behavioral impairments in male BDNF^+/−^ mice.

First, BDNF^+/−^ mice had deficits in the ASST. This task consists of several phases and starts with discrimination learning followed by several phases demanding cognitive flexibility, i.e., reversals and intra- and extradimensional shifts^42,80^. Using a similar behavioral paradigm in an operant chamber, impaired reversal learning was found previously in BDNF^+/−^ mice^50^. In the present study, BDNF^+/−^ mice had performance deficits throughout all phases of the ASST, i.e., the deficit was not restricted to reversal learning but also present in the other phases. Of note, such a general ASST performance deficit has been often observed in schizophrenic patients^81–83^. Our finding further supports human data showing that disruption of BDNF functioning leads to impaired cognitive flexibility^84,85^. Based on reports showing that interventions supporting BDNF function can facilitate and/or restore cognitive flexibility^86,87^, we housed our mice in EE that is known to increase BDNF brain levels^88^. We observed both expected EE effects: increased brain BDNF levels, as well as a rescue of the impairment in cognitive flexibility. To the best of our knowledge, such a complete rescue of a BDNF heterozygosity-induced deficit in cognitive flexibility by noninvasive restoration of BDNF brain levels back to normal through EE, has not been described before. However, Chourbaji et al. reported previously that EE can rescue behavioral changes of BDNF^+/−^ mice in anxiety, object recognition, and pain sensitivity^58^. These and the present findings raise the interesting possibility that the right dosage of EE might ameliorate BDNF-dependent deficits in other neuropsychiatric and neurological disorders.

Our second experiment was focused on startle reactivity. In humans, disrupted BDNF functioning leads to a decrease of the startle response^89^ and for schizophrenia patients, no changes in startle reactivity are described^90^. Here, we observed an increased startle reactivity in BDNF^+/−^ mice when intense startle stimuli were used. This is in line with previous studies showing an exaggerated startle response in BDNF^+/−^ mice^91,92^. It is known that BDNF plays an important role in inner ear development^93^ and that age-related hearing loss is accompanied by a reduction of BDNF in cochlear neurons^94^. Of note, the higher reactivity of BDNF^+/−^ mice to intense startle stimuli was prevented by EE housing. Importantly, these changes in startle reactivity were not caused by differences in body weight, since in this study body weight was neither affected by genotype nor by housing condition. An increased body weight in BDNF^+/−^ mice was repeatedly reported^58,69,95,96^, including previous studies of our group^51^. However, we observed in our breeding line that this body weight difference disappeared with repeated backcrossing to C57Bl/6J^53^.

More related to schizophrenia is the PPI deficit we observed in the BDNF^+/−^ mice, which is in line with recently published data^91^. PPI is an operational measure of sensorimotor gating that is deficient in schizophrenia^97,98^. However, polymorphisms interfering with BDNF secretion appear to be not sufficient to impair sensorimotor gating in humans^99^. Previously, no consistent effects of BDNF deficiency on PPI and pharmacologically induced PPI deficits were found in mice and rats^49,91,100,101^. In some studies, male BDNF^+/−^ mice were found to be more or less sensitive to treatments impairing PPI^49,92,101^. An explanation for these inconsistent effects on PPI could be the different ages of the mice during testing. In mature adult BDNF^+/−^ mice, we found robust PPI deficits in this study. Despite it was shown that central BDNF administration can rescue PPI deficits^102^ and that EE can increase BDNF levels^88^ and rescue other behavioral deficits in BDNF^+/−^ mice^58,103^, it was not tested so far whether PPI-related changes in BDNF^+/−^ mice can be rescued by EE. In the present study, we found no PPI deficit in BDNF^+/−^ mice anymore, if they were exposed for two months to EE.

Last, we also tested our animals on learned safety and learned contextual fear. Contextual fear was not affected in BDNF^+/−^ mice. This is in line with published findings^104^ while others described deficits^64^. Of note, we used a protocol with many US presentations that might rescue deficits observed with weaker conditioning protocols. Interestingly, learned safety, i.e., fear inhibition, was impaired in BDNF^+/−^ mice. However, it is important to note that safety learning was generally not very pronounced in the present study. Nevertheless, the observed safety learning deficit is in line with the one described in patients with schizophrenia^47,48^ and psychosis^105^. Again, EE housing rescued the observed deficit in our mice.

After completing the behavioral tests, we measured BDNF protein levels in different brain areas. It should be noted that the used ELISA for BDNF measurement has a limited cross-reactivity with pro-BDNF (ca. 13%), i.e., the measured levels are not exclusively mature BDNF. However, we generally observed that both experimental factors of the present study robustly affected BDNF levels: BDNF haploinsufficiency decreased and EE housing increased BDNF levels in all analyzed brain areas^38,88^. In some of the brain areas, the BDNF levels were well correlated with behavioral performance. In general, increased BDNF levels were associated with a rescue of the behavioral deficits in BDNF^+/−^ mice. However, the BDNF increase in BDNF^+/+^ mice did not affect behavioral performance, disregarding the mild improvement in reversal learning. This suggests that a certain threshold level of BDNF, which needs to be well above the 50% reduction found in BDNF^+/−^ animals, is required for intact functioning of the brain, while an additional increase of BDNF levels beyond this threshold does not further improve brain functioning^51,106^. However, this does not exclude that higher BDNF levels have protective effects on the future, e.g., age-related impairments^107^. Of note, too high, i.e., exaggerated BDNF levels, e.g., in BDNF-overexpressing mice, are also associated with behavioral impairments, including working memory deficits, PPI deficits, decreased startle, and increased anxiety^108^.

We observed correlations between BDNF brain levels and behavior here. Of note, such correlations do not necessarily indicate causality. However, it is not simple to experimentally prove that the mechanism of action for the rescue of behavioral deficits by enriched environment is the increase of BDNF brain levels. Potential experiments would be to “replace” enriched environment by chronic pharmacological activation of TrkB receptors. Such experiments have been performed in previous studies using either a TrkB receptor-activating antibody or the TrkB receptor agonist 7,8-dihydroxyflavone, and both manipulations were able to rescue memory deficits in different rodent models of human diseases such as schizophrenia^109,110^, depression^111^, Down syndrome^112,113^, Parkinson’s disease^114^, Hungtington’s disease^115^, and Alzheimer’s disease^116–119^ (but see also^120^). Another possibility would be to block the enriched environment effect by chronic application of a BDNF scavenger (e.g., TrkB-Fc). However, since the temporal and brain-wide spatial pattern of the enriched environment effect is not known yet, it is presently unclear whether such pharmacological interventions should use the chronic, subchronic, or acute application, and whether systemic vs. local, or continuous vs. intermittent regimes for activation of BDNF/TrkB signaling should be used. Future studies need to focus on a better understanding of the exact patterns of the enriched environment effects, which would then be a good basis for follow-up studies with the aforementioned pharmacological interventions.

In summary, we showed that BDNF haploinsufficiency in mature adult mice is associated with different behavioral endophenotypes of schizophrenia and that these endophenotypes are rescued by EE housing. However, our study has several limitations: first, we only tested male mature adult mice. Therefore, it is unclear whether the observed endophenotypes developed in a schizophrenia-typical way, i.e., after puberty, and whether the same can be observed in female mice. We focused on male mice since in some human studies a more important role of BDNF in schizophrenia was found in male patients^33,121^. Of note, many of the behavioral and neurochemical changes described in BDNF^+/−^ mice were only observed or more pronounced in male mice^49,101,122,123^. Second, we showed that EE housing increased BDNF brain levels and rescued the observed endophenotypes. However, despite it is very unlikely, these two effects of EE do not have to be associated with each other. It could be that the observed rescue was not mediated by the increase in BDNF levels, but another mechanism that is also influenced by EE (e.g., orexin A^124,125^). Third, it is unclear which aspect of EE was responsible for the rescue of behavioral deficits and the increase in BDNF levels, respectively. We suggest that wheel running during EE housing, i.e., voluntary exercise, plays a major role in increasing BDNF levels and rescuing the behavioral phenotypes. This is in line with numerous reports showing that physical exercise efficiently reduces psychiatric symptoms in schizophrenia and other neuropsychiatric disorders^126,127^.

## Supplementary information

Supplementary Material
