# Supplementary material for: BDNF haploinsufficiency induces behavioral endophenotypes of schizophrenia in male mice that are rescued by enriched environment
Source: Transl Psychiatry. 2021 Apr 22;11:233. doi: 10.1038/s41398-021-01365-z (PMC8062437; doi:10.1038/s41398-021-01365-z)
Supplement: Supplementary file 1 — Supplementary Material [file 41398_2021_1365_MOESM1_ESM.docx]

**Supplement**

**Protocol - Safety Learning and Contextual Fear Conditioning**


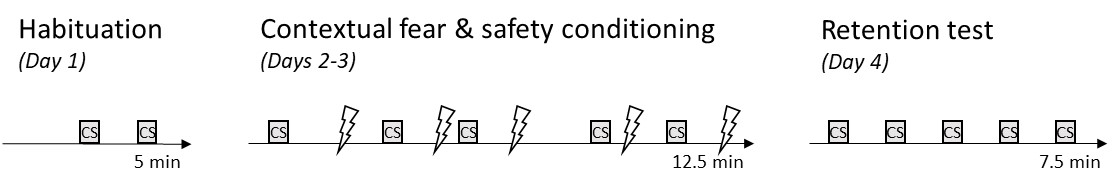


**SUPPLEMENTARY FIGURE 1** Protocol of the safety and contextual fear conditioning experiment. On day 1, the mice were habituated to the setup and the perspective safety CS. On day 2 and 3, five explicit unpairings of the CS and 5 electric stimuli with interstimulus intervals between 30 and 90 s were presented. On day 4, a retention test was performed.

**Attentional Set Shifting Task – Analysis of Enrichment Effects in BDNF^+/+^ Mice**

**SUPPLEMENTARY FIGURE 2** Enriched environment improve performance of BDNF^+/+^ mice in the different reversal phases. (A) Trials to criterion (mean + SEM) before reaching the criterion of six consecutive correct trials in the reversal phases of the ASST. (B) In BDNF^+/+^ mice, the BDNF levels in the FC were increased by exposure to enriched environment and correlate with the performance in the reversal phases.

**Attentional Set Shifting Task – Analyses of Errors**

The analysis of the errors until reaching the criterion of six consecutive correct trials confirms the analysis of the trials to criterion. In standard housed mice (Suppl. Figure 3A), there was a main effect of genotype (F_1,16_ = 19.66, p = 0.0004) and phase (F_5,80_ = 11.69, p < 0.0001) but no interaction (F_5,80_ = 1.01, p = 0.42). This genotype effect was not present in the mice previously housed in enriched environment (Suppl. Figure 3B; F_1,18_ = 1.41, p = 0.25) but there was still an effect of phase (F_5,90_ = 8.46, p < 0.0001) and no interaction (F_5,90_ = 0.63, p = 0.63). An ANOVA including the mean overall performance (Suppl. Figure 3C) revealed that there were significant main effects of genotype (F_1,34_ = 16.14, p = 0.0003) and housing condition (F_1,34_ = 14.39, p = 0.0006), as well as a significant interaction (F_1,34_ = 6.519, p = 0.02).

Taken together, this indicates that standard-housed BDNF^+/-^ mice generally made more errors in the ASST, an effect which was rescued by previously housing them in enriched environment.

**SUPPLEMENTARY FIGURE 3** Enriched environment rescues the performance deficits in the ASST in BDNF-haploinsufficient mice. (A-C) Number of errors before reaching the criterion of six consecutive correct trials in the ASST, either depicted for the single phases of the task (A-B) or for the overall performance, i.e. the mean of all phases (C). (A) In all phases of the task, the numbers of errors before reaching the criterion was increased in BDNF-haploinsufficient mice after standard housing demonstrating an overall impairment. (B) After enriched environment, this difference between the genotype was not observed (C) indicating that enriched environment rescued the impaired performance in BDNF-haploinsufficient mice. ** p < 0.01, main effects in ANOVA (A) or post-hoc comparisons as indicated (C).

**Analyses of Body Weight**

**SUPPLEMENTARY FIGURE 4** In the present study, the body weight of the animals was not affected by genotypes or housing. (A) Body weight at the end of the ASST, i.e. after food-deprivation (genotype: F1,34 = 0.0004; p = 0.99; housing condition: F1,34 = 0.12; p = 0.73; interaction: F1,34 = 0.02; p = 0.90). (B) Body weight was generally higher in non-food-deprived mice (overall ANOVA: F1,71 = 47.30; p = 0.01). However, again, genotype and housing condition had no effect (Fs < 0.006; ps > 0.94).
